# Efficacy of Locally Applied Flurbiprofen in Sore Throat Treatment: A Scoping Review

**DOI:** 10.3390/biomedicines13123035

**Published:** 2025-12-10

**Authors:** Emili Dragaš, Dejan Tomljenović, Ivan Rašić, Ivana Jurčić Čulina, Lucija Svetina, Goran Geber, Davor Vagić, Andro Košec

**Affiliations:** 1 Department of Otorhinolaryngology and Head and Neck Surgery, School of Medicine, University of Zagreb, 10000 Zagreb, Croatia; emili.dragas1@gmail.com (E.D.); irasic007@gmail.com (I.R.); davorvagic1@gmail.com (D.V.); 2 Department of Otorhinolaryngology and Head and Neck Surgery, University Hospital Center Sestre Milosrdnice, Vinogradska cesta 29, 10000 Zagreb, Croatia; dejan_tomljena@yahoo.com (D.T.); goran.geber1@gmail.com (G.G.); 3 Department of Otorhinolaryngology and Head and Neck Surgery, School of Dental Medicine, University of Zagreb, 10000 Zagreb, Croatia; 4 Department of Environmental Health Engineering, University of Applied Health Sciences, Mlinarska 38, 10000 Zagreb, Croatia; solertia.ivanajurcic@gmail.com (I.J.Č.); lucijasv@gmail.com (L.S.); 5 Department of Cardiac Surgery, University Hospital Center Zagreb, 10000 Zagreb, Croatia

**Keywords:** sore throat, flurbiprofen, pharyngitis, POST

## Abstract

**Background and Objectives:** Sore throat is a common symptom that, due to its heterogeneous etiology and diverse clinical presentation, poses challenges for both accurate diagnosis and etiological treatment, making symptomatic therapy essential. Our review aims to provide an overview of the literature on locally applied flurbiprofen in the treatment of sore throat of both infectious and non-infectious etiologies. **Materials and Methods:** The database PubMed was searched for studies evaluating the effect of flurbiprofen on sore throat in the last ten years (28 August 2025), and 15 studies were selected for the analysis based on the predefined inclusion and exclusion criteria. We analyzed the effect of flurbiprofen in acute pharyngitis on relief of pain, difficulty swallowing, throat swelling, the Qualities of Sore Throat Index, overall satisfaction scores and Upper Respiratory Tract Infection Questionnaire, stratified by single or multiple doses and concomitant antibiotics. Efficacy in postoperative sore throat treatment and safety profile were also analyzed. The results are demonstrated in tables and forest plots. **Results:** Topical flurbiprofen is shown to be effective in pain relief, relief of difficult swallowing and throat swelling in acute pharyngitis in both single and multiple doses, regardless of the formulations. The same effect is noted when analyzed by the Qualities of Sore Throat Index questionnaire and overall satisfaction scores. The data shows that flurbiprofen reduces related upper respiratory tract infection symptoms. It is also shown to be effective in reducing the incidence and severity of postoperative sore throat. No serious adverse effects were reported in the included studies. **Conclusions:** Topical flurbiprofen seems to be an effective and safe symptomatic treatment option for sore throat in both infectious and non-infectious etiologies.

## 1. Introduction

Sore throat, a symptom encompassing a painful, scratchy and burning sensation in the throat, is most often of infectious etiology, with viruses responsible for 90% of adult sore throats [1,2,3]. Group A ß-hemolytic streptococci are the most prevalent bacterial cause (5–36%) [2]. Sore throat can also have a non-infectious etiology, most often due to physico-chemical factors, such as smoking, snoring and shouting, or environmental factors, such as pollution, humidity or air conditioning [2]. It can also be a minor complication of intubation, expressed as postoperative sore throat (POST) and hoarseness as a result of trauma to the upper airway, resulting in inflammation, pain and altered function [2,4]. Sore throat, precisely acute pharyngitis, is most often self-limiting and does not produce serious consequences in otherwise healthy individuals [3]. It is, however, shown to be a recurring condition, causing distress and disruption of everyday life, due to discomfort, difficulty swallowing and sleeping disturbances, leading to a consequent lack of productivity and focus, additionally reported as one of the most frequent reasons to seek medical care [1]. It can be accompanied by fever, cough, swollen lymph nodes and hoarseness [1]. Due to its etiological heterogeneity and diverse clinical presentations, it is hard to adequately identify the cause and start targeted treatment [1]. Moreover, there are differences in treatment guidelines between regions and institutions, often leading to inappropriate antibiotic prescribing [1]. This is further encouraged by patients wanting quick relief and a lack of accurate diagnostic tools, leading to antibiotic overuse and antimicrobial resistance (AMR), even though the majority of cases are viral [1]. Therefore, effective and safe symptomatic treatment is of high importance. As a sore throat is a result of inflammation of mucous membranes in the oropharynx accompanied by the release of inflammatory mediators, specifically of prostaglandins (PGE_2_), topical non-steroidal anti-inflammatory drugs are the intuitive choice, with flurbiprofen showing rapid anti-inflammatory activity in vitro [5,6]. Flurbiprofen is a NSAID, demonstrating anti-inflammatory, antipyretic and analgesic properties, initially used in rheumatology [7]. Its use in acute inflammation of the upper respiratory tract started with systemic use in a study published in 1986, but then shifted to local administration in the treatment of sore throat in studies published in the early 2000s [8,9,10,11]. Flurbiprofen administered locally penetrates through all layers of the pharynx mucosal tissue and reaches the lamina propria, which contains blood vessels and nerve fibers contributing to pain [6]. There are many studies investigating the efficacy and safety of flurbiprofen in the symptomatic treatment of sore throat, mostly in the context of URTIs, with fewer studies investigating its effect in non-infectious cases. The aim of our scoping review is to provide an overview of the literature on flurbiprofen in the treatment of sore throat of both infectious and non-infectious etiologies, focusing on different clinical features such as pain, difficulty swallowing and throat swelling, while also assessing its effect on other URTI symptoms and overall safety, intending to highlight possible research gaps and provide objective conclusions to further guide concrete treatment recommendations.

## 2. Materials and Methods

This scoping review was conducted in accordance with the PRISMA guidelines for scoping reviews, and the PRISMA 2020 checklist is included in the Supplementary Table S1. The scoping review was registered in the OSF database registry htpps://osf.io/mcb6d (accessed on 23 October 2025). The database PubMed was searched. In the initial search, the search terms flurbiprofen, sore throat and acute pharyngitis were used. The initial search revealed 49 articles (Table 1). The results were then limited to articles published in the last 10 years to ensure the most up-to-date results. This search revealed 30 articles in total. An additional language filter was applied, limiting the search to articles published in English. This final search, performed on 28th August 2025 at 10:20 am, revealed 27 articles that were considered in the further screening process.

The identified publications had to then be screened according to the PICOS defined in Table 2, demonstrating the inclusion and exclusion criteria for the studies considered for this scoping review.

**Figure 1:**
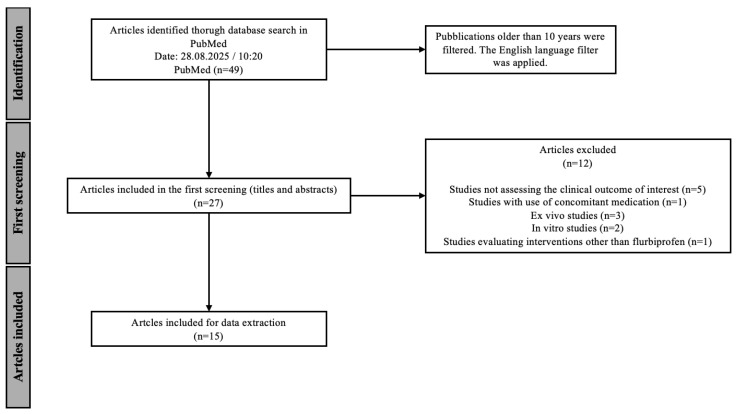
Flow chart of the screening process according to the PRISMA guidelines.

The entirety of the screening process is demonstrated graphically in Figure 1. In the initial screening, titles and abstracts were analyzed and 12 studies were excluded based on the exclusion criteria shown in Table 2. Due to the aim of this scoping review, five articles examining outcomes other than pain relief and overall symptomatic relief of sore throat were excluded, some of which were limited to the pharmacokinetics and pharmacodynamics of flurbiprofen and not the clinical outcome studied in this scoping review. Studies conducted in vitro or ex vivo (*N* = 5) were also excluded, as well as studies using other interventions (*N* = 1) or concomitant medication (*N* = 1). At the end of the screening, 15 articles were chosen for data extraction. When multiple publications reported data from the same clinical trial, these were considered as a single study for the purpose of participant counting, while relevant additional analyses were extracted separately. The results of the analysis are presented in tables and figures.

The chosen papers were evaluated using the Oxford Level of Evidence Guideline https://www.cebm.ox.ac.uk/resources/levels-of-evidence/ocebm-levels-of-evidence (accessed on the 29 August 2025). This guideline provides a framework for ranking the quality and reliability of research papers, dividing them into five categories. Level V represents the lowest level, papers based on mechanics-based reasoning, while Level I represents the highest level of evidence, mostly reserved for systematic reviews. The effect size of the studies, as well as the overall quality, precision and consistency, influence the ranking of papers.

Additionally, conflicts of interest and funding of the studies were noted due to the possible bias of results.

## 3. Results

The Results section is organized into four parts: an overview of the study cohort, an analysis of study outcomes in acute pharyngitis and POST, an evaluation of the safety profile of flurbiprofen and an overview of the included papers.

### 3.1. Study Cohort Overview

An overview of the study cohort of the included papers is presented in Table 3 and Table 4. Across the included clinical trials, a total of 1615 participants were analyzed. Review articles were not included in the participant count, as they summarize data from previously published studies, and the participant total is based on the analyzed population to avoid overestimation. The participants of the studies were mostly individuals with a sore throat. The majority of studies focused on individuals with acute pharyngitis, either with a confirmed upper respiratory tract infection (URTI) [12,13,14,15,16,17,18,19] or a diagnosed Streptococcal infection [12,20]. Two systematic reviews evaluating adverse effects included studies in which some of the participants were healthy volunteers [21,22]. Three of the analyzed papers used participants with postoperative sore throat [4,23,24]. The initial evaluation of participants was performed using different questionnaires and scales. The evaluation of the presence of an URTI was performed with the URTI questionnaire [12,13,14,16,17,18,20], an objective assessment of acute pharyngitis with the Tonsillo-Pharyngitis Assessment (TPA) [12,13,14,15,16,17,18,19,20], throat pain and soreness with the Sore Throat Scale (STS) [12,14,15,23], Throat Soreness Scale (TSS) [15,19,23], Throat Pain Scale (TPS) [12,13,14,16,18,20] and Sore Throat Pain Intensity Scale (STPIS) [12,13,16,17,18,20,23], difficulty swallowing with the Difficulty Swallowing Scale (DSS) [13,15,16,17,18,19,20] and throat swelling with the Swollen Throat Scale (SwoTS) [16,17,18,19,20], and a Practitioner’s Assessment of Inflammation (PAIN) [12,18] was also used. All the clinical trials excluded patients with mouth breathing, severe coughing or other respiratory illnesses that could precipitate a sore throat other than the initial diagnosis [12,13,14,15,16,17,18,19,20]. Participants with known allergies and contraindications to the tested drug were also excluded from the clinical trials. Patients using topical ‘’cold medication’’ in the 1–2 h time period prior to the administration of flurbiprofen or with a recent use of cold/influenza medication systemically were also excluded [12,14,15,16,17,18,20]. Recent antibiotic use was the exclusion criteria in four clinical trials [12,14,17,20].

All the studies included adult patients (≥18 years old), except for one included study in a systematic review [21] and one in a narrative review [23], where the study participants had to be at least 12 years old. None of the studies differentiated the results based on sex, with the distribution of male and female participants in the randomized control trials demonstrated in Table 3.

All the included papers used flurbiprofen in the treatment or prevention of sore throat. The studies used 8.75mg of flurbiprofen to treat acute pharyngitis, while different dosages were used in the studies evaluating its effect in the alleviation and prophylaxis of POST [4,24]. In all of the papers, flurbiprofen was used locally, in the form of sprays [13,15,16,19,21,23,24], lozenges [12,13,14,16,17,18,20,21,22,23] or microgranules [23]. One paper analyzed the use of flurbiprofen in combination with antibiotics in the treatment of Streptococcal pharyngitis [20]. Antibiotics were also used in the treatment of proven bacterial infection in the study published by the same author in 2018 [12]. Some of the studies allowed participants to take paracetamol tablets, for additional analgesia if needed, for the duration of the study [12,14,17,18,19]. The majority compared flurbiprofen with a placebo [4,12,14,15,17,18,19,20]. Flurbiprofen was compared with a placebo and a benzydamine hydrochlorine spray in the prevention of POST [24]. Two studies compared the same dosage of flurbiprofen but in different formulations, lozenge and spray [13,16]. These two studies, in fact, use the same clinical trial, but only differ in the measured outcomes. This was the case in two other instances, with de Looze et al. [15,19] and Schachtel et al. [12,14] using the same clinical trial in two different papers and analyzing different outcomes. The included narrative review examined studies in which all the aforementioned comparisons were made, along with studies in which flurbiprofen was compared with sea salt/glycerine spray and a stomatitis/gingivitis gargle or a dosage of 8.75mg was compared to 12.5mg [23].

### 3.2. Outcomes After Flurbiprofen Use in Acute Pharyngitis

Based on the analyzed data, we report the efficacy of flurbiprofen on sore throat in acute pharyngitis with respect to the reduction in pain, difficulty swallowing and throat swelling, followed by evaluation with the composite index QuaSTI and overall treatment ratings. Additionally, the effect of flurbiprofen on URTI symptoms was assessed. All evaluations were conducted using questionnaires and scales, which are listed in Table 5. Each outcome was further stratified by relevant subcategories (single/multiple doses, concomitant antibiotic use).

#### 3.2.1. Pain and Soreness Relief

Changes in pain and soreness levels were analyzed in eight clinical trials, one review and one consensus document (Table 6). A single dose of flurbiprofen was compared with placebo in four studies [12,15,18,19], with two of them additionally assessing the effect after multiple doses [18,19] and one assessing multiple doses only [17]. Lozenges were applied in four trials [12,17,18,20] and sprays in two trials [15,19]. These two formulations were compared in two papers [13,16]. A concomitant use of antibiotics was analyzed in one paper [20]. Assessments were conducted using STPIS, STRRS, TSS and STS, with one clinical trial additionally employing the DSW method [12]. All mentioned formulations and dosages were analyzed in the narrative review [23]. Overall, clinical trials showed a significant effect of 8.75 mg of flurbiprofen in reducing throat pain and soreness (Table 6), also presented in the narrative review [23] and achieving a 100% degree of agreement in a consensus published by Abdullah et al. in 2024 [1].

**Figure 2:**
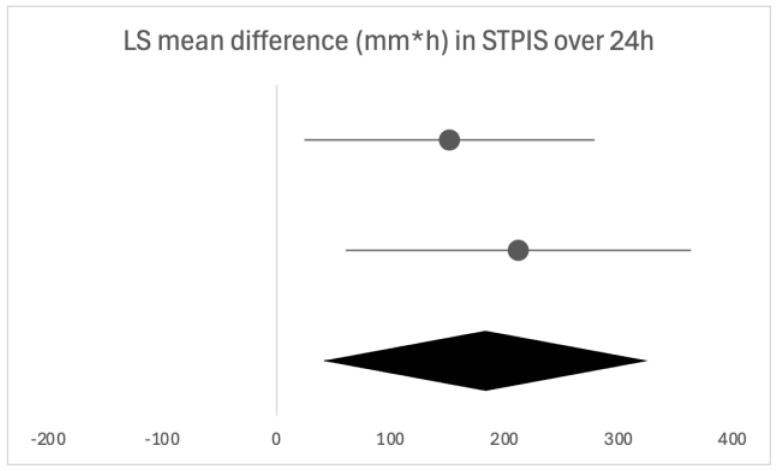
Forest plot of the two eligible studies for the time-weighted LS mean difference in STPIS after 24 h of flurbiprofen or placebo dosing. The horizontal line above is quoted from [13], the horizontal line below is quoted from [14].

Due to the heterogeneity of the studies, only two were eligible for a forest plot [17,18] presented as Figure 2.

##### Single Dose

Statistically significant greater pain relief was reported by participants after receiving only one dose of flurbiprofen compared with placebo in all of the studies (Table 6).

Time to first perceived pain relief with flurbiprofen was 11 min, as reported by Schachtel et al. (DSW) [12], while significantly greater pain relief than placebo was reported at the 5 min mark (TSS) [19] and the 20 and 22 min mark (STPIS, STRRS) [15,18,19]. Meaningful pain relief was reported 43 min after dosing (DSW) [12]. The duration of pain relief was significantly greater with flurbiprofen than with placebo for the duration of the follow-up period: six hours in two papers [15,19] and three hours in one paper [12]. A trial by Aspley et al. also had a follow-up time of six hours but reported a significant reduction in pain for only 3.5 h [18]. When analyzing the extent of pain reduction, 78% of participants in the flurbiprofen group reported meaningful pain relief (*p* < 0.01) in the study by Schachtel et al. [12]. De Looze et al. [19] showed that 55% of participants in the flurbiprofen group reported at least 30 min of at least moderate relief (measured by STRRS) in the six hours following flurbiprofen administration (*p* < 0.0001). This was then confirmed in the subgroup analysis of this clinical trial published in 2018 by the same author [15].

There was no statistically significant difference between the flurbiprofen formulations (spray vs. lozenge) in the effectiveness of pain reduction (Table 6) [13,16]. Burova et al. [13] reported that 74–78% of participants had at least moderate pain relief two hours after dosing with flurbiprofen, but, as there is no control group, it is not possible to conclude the effectiveness of flurbiprofen itself in this particular study. These results are, however, similar to those where a control group was included (Table 6).

##### Multiple Doses

When assessing the effectiveness of multiple doses of flurbiprofen on pain relief, three clinical trials reported on its efficacy [17,18,19]. After 24 h, there was a 47% (*p* < 0.05) [17] and a 79.8% (*p* < 0.01) [18] greater mean reduction in pain compared with placebo, measured with STPIS, further shown in Figure 2. After a three-day follow-up period, de Looze et al. [19] reported that flurbiprofen provided a greater change from baseline measures than placebo at the end of each of the three days (measured by TSS, STPIS and STRRS, all *p* < 0.05). The longest follow-up time was seven days and there was a 74% greater pain reduction before and two hours post-dose in the flurbiprofen group from days two to seven compared with the placebo group (STPIS, *p* < 0.01) [17]. A reduction in pain with flurbiprofen was also reported in the consensus document [1] (Table 6).

##### Concomitant Antibiotics

The only study that focused on the use of flurbiprofen with antibiotics was by Schachtel et al. [20]. They found that pain relief was 93% greater in the flurbiprofen group compared with placebo (*p* = 0.05) in the 24 h before antibiotic administration and 84% during antibiotic administration (*p* = 0.04). They concluded that antibiotics did not influence the pain relief outcomes of flurbiprofen (*p* = 0.96).

#### 3.2.2. Difficulty Swallowing

Five clinical trials directly evaluated changes in difficulty swallowing following flurbiprofen administration. Three of them compared flurbiprofen with placebo [17,18,19], with two reporting outcomes after both single and multiple doses [18,19], while one reported outcomes only after multiple doses [17]. One narrative review [23] and one consensus document [1] reported on the changes in difficulty swallowing. One paper compared flurbiprofen formulations, spray and lozenge [13], and one combined it with antibiotics [20]. Difficulty swallowing was assessed by the DSS in all of the included studies (Table 7). Overall, flurbiprofen was shown to have a beneficial effect on difficulty swallowing after both single and multiple doses (Table 7).

**Figure 3:**
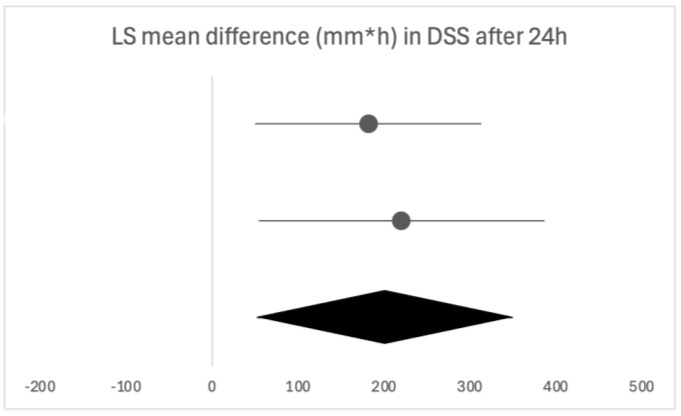
Forest plot of the two eligible studies for the time-weighted LS mean difference in DSS after 24 h of flurbiprofen or placebo dosing. The horizontal line above is quoted from [13], the horizontal line below is quoted from [14].

Due to the heterogeneity of the studies, only two were eligible for a forest plot [17,18], presented in Figure 3.

##### Single Dose

Greater changes from baseline in difficulty swallowing after flurbiprofen administration compared with placebo were first noticed five minutes after administration in a study by de Looze et al. [19] and ten minutes by Aspley et al. [18]. It is worth noting that Aspley et al. [18] administered flurbiprofen lozenges and did not specify whether this was noted ten minutes after placing the lozenge in the mouth or after its complete dissolution. Both studies had a follow-up time of 6 h, with one reporting the duration of the effect as 3.5 h and one for the full 6 h [14] and [15], respectively]. Regarding the extent of relief, concrete reports were not provided.

When comparing different flurbiprofen formulations, spray and lozenge, Burova et al. [13] found no statistically significant difference in the reduction in difficulty swallowing between the two formulations, with both formulations providing a change from baseline (Table 7).

##### Multiple Doses

After the administration of multiple doses of flurbiprofen over 24 h, flurbiprofen was shown to provide a greater mean improvement than placebo, 66% by Schachtel et al. (*p* < 0.01) [17] and 9.6% by Aspley et al. (*p* < 0.01) [18]. These results are also shown in Figure 3. Similar results were reported in the consensus document by Abdullah et al. [1] (Table 7). Over the course of three days, de Looze et al. [19] reported a greater change from baseline with flurbiprofen than placebo at the end of each day (all *p* < 0.05). The longest follow-up period was presented by Schachtel et al. [17], where there was a 72% greater mean improvement in swallowing with flurbiprofen before and two hours post-dose in days two to seven (*p* < 0.01).

##### Concomitant Antibiotics

When flurbiprofen was coadministered with antibiotics, there was a 107% greater reduction in difficulty swallowing than with placebo (*p* = 0.04), but no statistically significant difference was found between flurbiprofen and placebo in the 24 h before antibiotic administration (Table 7). Schachtel et al. [20] concluded that antibiotics alone provided no significant relief of difficulty swallowing.

#### 3.2.3. Throat Swelling

Of the included studies, seven documented the effect of flurbiprofen on throat swelling (Table 8). Flurbiprofen was compared with placebo in three clinical trials [17,18,19], with all three reporting the effect after multiple doses of flurbiprofen and two additionally providing data after a single flurbiprofen dose [18,19]. The included narrative review [23] reported the effects after both dosing regimens and using all the mentioned formulations, while the consensus document mentioned only flurbiprofen lozenges in multiple doses [1]. Flurbiprofen formulations, spray and lozenge, were compared in one study [13], and one study additionally treated patients with antibiotics [20]. Throat swelling was assessed by SwoTS (except for [1]) (Table 8). Overall, flurbiprofen was generally found to be effective in reducing the sensation of throat swelling after both single and multiple doses (Table 8).

**Figure 4:**
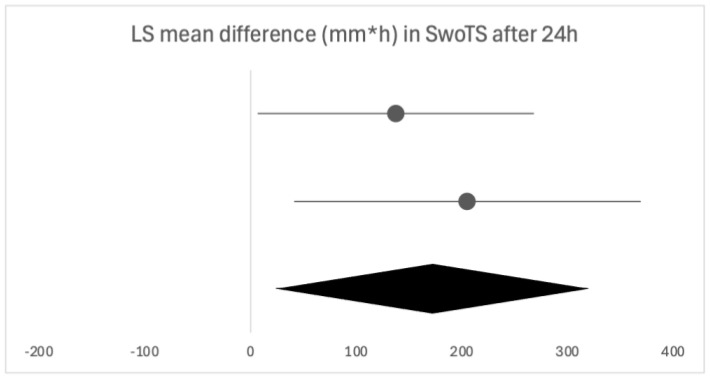
Forest plot of the two eligible studies for the time-weighted LS mean difference in SwoTS after 24 h of flurbiprofen or placebo dosing. The horizontal line above is quoted from [13], the horizontal line below is quoted from [14].

Due to the heterogeneity of the studies, only two were eligible for a forest plot [17,18], presented in Figure 4.

##### Single Dose

In the two studies presenting data after a single dose of flurbiprofen, the first significant relief of throat swelling compared to placebo was achieved after 30 min [19] and 60 min [18]. These were the first assessment times, so it remains unknown whether the effect would have been present earlier after dosing. Aspley et al. [18] reported that the duration of the effect was 180 min, and de Looze et al. [19] reported six hours.

No difference was found between flurbiprofen 8.75 mg spray and lozenge in the relief of throat swelling at one and two hours post-dose, with both of the formulations showing changes from baseline in favor of flurbiprofen (Table 8) [13].

##### Multiple Doses

Multiple doses of flurbiprofen over 24 h led to a greater mean reduction in the sensation of throat swelling compared with placebo, 40% [17], 44% [1] and 69.3% [18]. When assessed at later time points, Schachtel et al. [17] reported a 63% greater reduction in swelling before and two hours post-dose in days two to seven, while de Looze et al. [19] reported a significantly greater reduction at the end of days one and two but not at the end of day three (*p* = 0.147).

##### Concomitant Antibiotics

In the study by Schachtel et al. [20], there was a 295% greater reduction in the sensation of throat swelling with flurbiprofen compared to placebo 24 h before antibiotic coadministration (*p* = 0.008). After antibiotic coadministration, there was a 70% greater reduction, although not statistically significant (*p* = 0.06). In the flurbiprofen group, antibiotics did not influence throat swelling (Table 8).

#### 3.2.4. Other Scores (QuaSTI)

QuaSTI, an index composed of words and phrases commonly used by patients to describe a sore throat, was used as an assessment tool in two clinical trials [13,14] and one narrative review [23]. After a single dose of flurbiprofen lozenge, there was a significantly greater change from baseline of the overall score than after the placebo (*p* < 0.01) [14]. When individual points of the index were analyzed, flurbiprofen resulted in a greater mean change from baseline than placebo in almost all points (Table 9). Burova et al. [13] compared the flurbiprofen spray and lozenge and found no significant difference between the formulations, as both resulted in improvement for each score of the index two hours after dosing (*p* < 0.0001). As the narrative review included the same two trials, a similar conclusion was made [23] (Table 9).

#### 3.2.5. Overall Scores

Overall satisfaction with treatment was rated in four clinical trials [15,16,17,19] and one narrative review [23]. Patient-oriented scales, SATIS and GLOBAL, were used in all four papers, while a practitioner’s evaluation, CLIN, was used in three papers [16,17,19]. Overall, significantly more patients taking flurbiprofen and practitioners rated the treatment as ‘’good’’ or were at least ‘’satisfied’’ compared with a placebo after both single and multiple doses (Table 10).

##### Single Dose

Following a single dose, de Looze et al. [15] reported that three hours after dosing, 53.2% of patients treated with flurbiprofen were at least ‘’satisfied’’ with the treatment, significantly more than the placebo group (SATIS, *p* < 0.0001). At the two-hour mark, 54% of patients treated with flurbiprofen rated the treatment as at least ‘’good’’ on the GLOBAL scale (GLOBAL, *p* < 0.01) [17].

When comparing formulations, there was not a significant difference between the flurbiprofen spray and lozenge in overall satisfaction scores [13,16] (Table 10).

##### Multiple Doses

Over 24 h, significantly more patients rated the treatment as at least ‘’good’’ (GLOBAL) or were at least ‘’satisfied’’ (SATIS) in the flurbiprofen group than the placebo (all *p* < 0.01) [17]. When CLIN was used, more practitioners rated the treatment with flurbiprofen as at least ‘’good’ compared to the placebo, both at the end of day one and day seven (all *p* < 0.01) [17]. When evaluated at the end of day three, the results were similar [15,19] (Table 10).

#### 3.2.6. URTI Questionnaire

While a lot of the clinical trials used the URTI questionnaire in the initial assessment, only two used it as an assessment tool after flurbiprofen use [13,14]. Overall, flurbiprofen was shown to decrease the number of URTI symptoms (Table 11).

The paper by Schachtel et al. [14] found that there was a greater change from baseline three hours after flurbiprofen administration compared to placebo, where no significant change was noted. In 46% of the patients who reported coughing at the beginning of the study, there was no coughing present three hours post-dose [14]. Although Burova et al. [13] did not compare the results with placebo, they did report a decrease in the number of patients experiencing URTI symptoms related to ST two hours after dosing. They also reported that a number of patients developed throat tickle and clearing and explained it as a possible common side effect of local flurbiprofen administration (throat irritation) [13]. The effect on URTI symptoms was also assessed in a narrative review by de Looze et al. [23], where they confirmed the beneficial effect of flurbiprofen compared with placebo.

### 3.3. POST

The efficacy of flurbiprofen in the prevention of POST was analyzed in two clinical trials [4,24] and one narrative review [23]. Calabrese et al. [4] analyzed the effect of a flurbiprofen solution applied in the endotracheal tube ten minutes after intubation, ten minutes after ICU admission and ten minutes before extubation, and found that almost all patients with flurbiprofen had no POST or hoarseness in the 36h following extubation, compared with placebo (*p* < 0.001) (Table 12). A smaller incidence and severity of POST was also shown by Muderris et al. [24], in whose study flurbiprofen was administered before intubation (Table 12). A narrative review by de Looze et al. [23] analyzed the effect of a single dose of flurbiprofen prior to intubation and found similar results in the early postoperative period [23] (Table 12). Flurbiprofen was also shown to lead to a higher degree of patient satisfaction than placebo (*p* < 0.001) [4]. Therefore, flurbiprofen administered before or after intubation was demonstrated as effective in preventing and reducing the severity of POST (Table 12).

### 3.4. Safety Profile of Flurbiprofen

The safety profile of flurbiprofen was assessed in 14 studies (Table 13). The incidence of any adverse effects (AEs) varied between papers, but AEs related to flurbiprofen were reported in four clinical trials and two systematic reviews (Table 13). They were most commonly related to the digestive tract (nausea, dyspepsia, diarrhea, abdominal pain/discomfort) and nervous system (dry mouth, paresthesia, throat irritation), as reported in a narrative review by de Looze et al. [23]. Serious AEs and AEs leading to discontinuation were rare and generally related to underlying medical conditions or later on shown not to be of clinical relevance [23] (Table 13). Evans et al. [21] specifically examined AEs resulting from drug interactions and found that no such AEs were reported. Dhanda et al. [22] found limited evidence on the risk of hemorrhagic events with the use of 8.75 mg of flurbiprofen, but did not exclude the possibility of its occurrence. The consensus by Abdullah et al. [1] received a 100% degree of agreement that topical NSAIDs (flurbiprofen) have a better benefit-to-risk ratio than oral NSAIDs. They also mentioned that topical NSAIDs do not induce GI AEs and risk of renal failure [1]. In general, flurbiprofen 8.75 mg applied locally can be considered a safe option for the symptomatic treatment of sore throat (Table 13).

### 3.5. Paper Review

**Figure 5:**
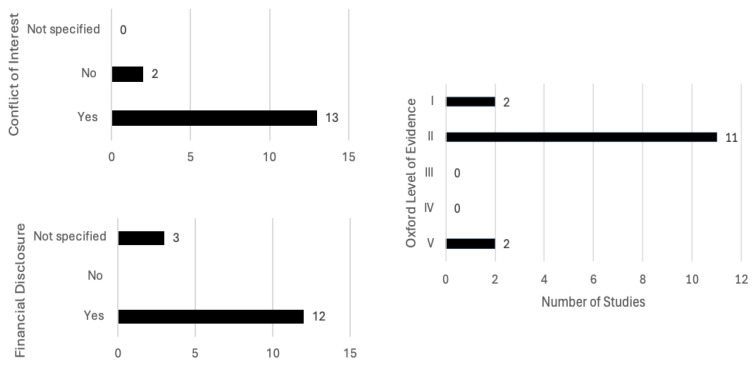
Scoping reviews included papers’ quality evaluation using the Oxford Level of Evidence, additional disclosure of financial assistance and stated conflicts of interest.

Studies included in this review were ranked high on the Oxford Level of Evidence, as there were two studies of the highest Level I, eleven of Level II, and only two studies of the lowest Level V (Figure 5). A large proportion of studies (11/15, 73.3%) were funded by Reckitt Benckiser Healthcare Ltd., UK, with the majority of studies stating a conflict of interest due to employment in the company (12/15, 80%). As Reckitt Benckiser is a manufacturer of flurbiprofen lozenges and sprays, this should be considered when interpreting the results of the papers. A financial disclosure was not specified in three of the studies, although in one of them, Reckitt Benckiser funded medical writing assistance, and a conflict of interest due to employment at Reckitt Benckiser was stated [19]. In the papers focused on POST, there was no reported conflict of interest.

## 4. Discussion

In this scoping review, we sought to provide an overview of the current literature on the effectiveness of topical flurbiprofen in the management of sore throat, including both infectious and non-infectious etiologies, precisely acute pharyngitis and POST. Most studies to date have focused on acute pharyngitis, while significantly fewer studies have investigated its role in POST. Our analysis concluded that locally applied flurbiprofen 8.75 mg appears effective in alleviating all key clinical features of sore throat in acute pharyngitis (throat soreness, difficulty swallowing, throat swelling) and in the prevention of POST. In our review, we evaluated locally applied flurbiprofen without stratifying findings by formulations, as two included studies reported no differences between the spray and lozenge across all clinical features, thus allowing the choice to be guided by individual preference. Similar results to ours were presented in a narrative review published by de Looze et al. in 2019, where they concluded that flurbiprofen is a useful first-line treatment option for symptomatic relief in sore throat associated with URTI and as a preoperative treatment for reduction in early POST [23]. In the included papers, sore throat was evaluated by different questionnaires, all focused on different aspects of the broad term; hence, we divided our analysis accordingly. Pain relief was the only symptom evaluated with multiple questionnaires, all of which ultimately yielded consistent conclusions. As the results are reported heterogeneously, we performed a qualitative analysis and presented all the available evidence and conclusions in the current literature.

Firstly, we will discuss the effect of flurbiprofen in acute pharyngitis. When overall satisfaction with the treatment was evaluated by patients and clinicians, the satisfaction rate was significantly higher with flurbiprofen than with placebo. The onset of action of flurbiprofen is quick, with significant pain relief occurring as early as five minutes and 22 min at the latest following administration, especially important due to the distressing nature of the symptom. Similar results were noted in the assessment of difficulty swallowing. Throat swelling decreased in the first assessments, which were performed later compared to the other two parameters, at 30 and 60 min, leaving room for discussion as to whether the actual onset time is earlier than assessed. The instructions are to take flurbiprofen every three to six hours, as needed, which is in line with the findings in our review, as the duration of the effect of a single dose was three to six hours, depending on the follow-up time [25]. Acute pharyngitis typically resolves within three to seven days, with symptoms sometimes persisting for up to ten days; hence, multiple doses of flurbiprofen are required in its treatment, and this aspect was also examined in the included studies [6,26]. Following the usual clinical course, seven days was the longest assessment time, where flurbiprofen was shown as effective in all of the analyzed clinical features. The same conclusion can be drawn from studies opting for shorter follow-ups of 24 to 72 h, confirming the beneficial effects of flurbiprofen after multiple doses. While the majority used multiple questionnaires to assess different clinical features, an alternative is QuaSTI, a composite index including all of the mentioned clinical features, divided into three factors, which was used as an assessment tool in three of the studies. This index is said to be useful in the direct comparison of analgesic products for sore throat, possibly enabling a more direct comparison [27]. The results in the included studies were consistent with the findings from the individual evaluations, making it a good comprehensive assessment tool. As acute pharyngitis is often part of an upper respiratory tract infection, accompanied by other relevant clinical symptoms, we examined whether the effect of flurbiprofen went beyond the relief of a sore throat and had an effect on the other accompanying symptoms in a URTI. Flurbiprofen was shown to provide an overall greater relief of these symptoms than placebo. It is important to note, however, that topical flurbiprofen provides symptomatic relief primarily through local effects, rather than systemic, as stated by a paper published in 2023 examining the relationship between the pharmacokinetic profile and clinical efficacy data of flurbiprofen [28]. It is possible that some of the questioned symptoms, such as lack of energy, loss of appetite, coughing and mouth breathing, were a consequence of the sore throat and had therefore disappeared following sore throat relief with flurbiprofen. Another thing to note is that most of the assessments on URTI symptoms were performed after a single dose of flurbiprofen, leading us to question whether a complete resolution of these symptoms could be accomplished in such a short time frame, as the symptoms are usually present for up to ten days [26]. There is an opportunity for future research to explore whether topical flurbiprofen has a direct effect on the duration of URTIs accompanied by sore throat. This would provide insight into its potential to not only relieve symptoms but also to influence the overall course of the infection.

As stated in the introduction, an important motive for the investigation of the effects of flurbiprofen on sore throat is the inappropriate use of antibiotics and the development of antimicrobial resistance, as the majority of adult cases of acute pharyngitis are of viral origin [1]. That said, in cases where bacterial etiology is confirmed, antibiotics are an essential part of treatment [20,26]. One of the included studies investigated the effects of flurbiprofen both before and during antibiotic administration in bacterial pharyngitis and found that antibiotics alone did not provide a significant relief of symptoms in the 24 h assessment time. This points to the conclusion that while antibiotics are essential in the treatment of bacterial pharyngitis, as they treat the cause itself, additional symptomatic therapy, such as flurbiprofen, should be provided to patients to alleviate symptoms, especially in the initial stages of treatment.

When assessing the effect of flurbiprofen in non-infectious etiologies of sore throat, only POST was investigated. Flurbiprofen was shown as effective in preventing and reducing the severity of POST when administered both before and after intubation (before extubation). This was accompanied by greater satisfaction of patients than with placebo and a smaller incidence and severity of hoarseness post-intubation. Since there are no studies investigating the effect of flurbiprofen on other non-infectious causes of sore throat, further studies are needed in this area. As pain and discomfort in a sore throat are a result of a release of inflammatory mediators, even in non-infectious causes of sore throat, it is reasonable to anticipate the beneficial effect of flurbiprofen even in these instances [6].

As with any treatment, safety should be a top priority, especially in symptomatic treatment and over-the-counter medication. Therefore, almost all of the included studies examined the safety profile of flurbiprofen. Topical flurbiprofen is shown to be a safe option for the symptomatic relief of sore throat, as no serious adverse effects were reported, and the overall number of treatment-related adverse effects was low. Local NSAIDs were shown to be a safer option than oral NSAIDs in a consensus document by Abdullah et al. [1], with all of the other included studies pointing to the efficacy of these treatment options, making them an overall better choice in the symptomatic relief of a sore throat. Since none of the included studies involved children under the age of 12, there is a substantial gap in evidence for the pediatric population. There is some data of orally administered flurbiprofen, but the data is nevertheless scarce, as mentioned in the 2022 review [25]. A study published in 2023 reported on the use of orally administered flurbiprofen for perioperative analgesia in children, reporting its safety and effectiveness [29]. However, due to the different route and context of administration, its findings cannot be directly applied to our analysis.

Due to the uniform results demonstrating flurbiprofen as a safe and effective symptomatic treatment option for sore throat, its addition to clinical guidelines could be considered. More studies are still needed to investigate its effect on non-infectious causes of sore throat, as only POST is covered in the current literature.

In these studies, patients were generally observed under optimal conditions, for example, being instructed to refrain from oral intake several hours after administration or having the spray applied by medical staff. Such conditions are unlikely to be consistently maintained in at-home settings, which should be considered in the interpretation of these results, as the clinical effectiveness may therefore be less pronounced.

One of the limitations of this review is patient selection, as most of the participants were young adults, with no representation of the pediatric population, one with a high incidence of acute pharyngitis, in the included studies. This underscores the need to investigate the effectiveness and safety in children. Limited safety data in adults and the inherent challenges of conducting a pediatric clinical trial may account for the lack of studies, yet additional research in this population remains crucial.

Even though safety was evaluated in a large number of studies, only a small number were specifically focused on investigating the safety profile. Further data is needed to confirm the absence of drug–drug interactions and risk of hemorrhagic events, due to the low security of the current evidence. Due to the heterogeneity of clinical features and a lack of a gold standard diagnostic tool, a secure confirmation of etiology is less likely, making the further synthesis of results more difficult. Another limitation is the lack of studies investigating non-infectious etiologies other than POST, limiting the findings to acute pharyngitis and POST.

## 5. Conclusions

According to the current body of literature, flurbiprofen applied locally is a safe and effective option for symptomatic relief of all of the clinical features of sore throat and for preventing and decreasing the severity of POST. Flurbiprofen appears to have a rapid onset of action and a sustained effect, with consistent outcomes reported for both the spray and lozenge formulations.

Evidence supporting the safety of locally administered flurbiprofen remains limited, particularly in the pediatric population, highlighting the need for appropriately designed studies. Moreover, the results across studies are presented heterogeneously, using varying assessment methods, which hinders uniform analysis and comparison.

## Figures and Tables

**Table 1:** Search terms and outcomes of each step of the search in the PubMed database.

Search Steps	Search Terms	Hits
1	(flurbiprofen) AND ((sore throat) OR (pharyngitis))	49
2	Filter: a time frame of the last 10 years	30
3	Filter: English language	27

**Table 2:** Inclusion and exclusion criteria for the identified publications.

**Inclusion Criteria**	
Population	Subjects of any age, gender or ethnicity with a sore throat (infectious and non-infectious etiology)
Intervention	Locally applied flurbiprofen (spray, lozenge, microgranules)
Comparator	Placebo or other pharmacological interventions
Outcomes	Symptomatic relief (pain relief, swallowing difficulty, throat swelling, hoarseness, URTI symptoms)
Study Design	Systematic reviews, meta-analyses, randomized control trails, case series, reviews, narrative reviews
**Exclusion criteria**	
	Studies not assessing the clinical outcome of interest Ex vivo studies In vitro studies Studies with use of concomitant medication Studies evaluating interventions other than flurbiprofen

URTI: Upper Respiratory Tract Infection.

**Table 3:** An overview of the study cohort in the included clinical trials.

Studies	Number of Subjects (*n*)	Male (*n*)	Female (*n*)	Age Average (in years)	Age Min (in years)	Age Max (in years)	Sore Throat	Intervention	Comparator	Aim
Calabrese et al., 2024 [4]	Total = 68; flurbiprofen = 34, placebo = 34	Total = 57; flurbiprofen = 29, placebo = 28	Total = 11; flurbiprofen = 5, placebo = 6	Total = 70; flurbiprofen = 69, placebo = 70	64 (63 for flurbiprofen, 65 for placebo)	73	POST (Postoperative Sore Throat)	Flurbiprofen 0.25% solution in subglottic part of the endotracheal tube	Placebo (saline)	Efficacy of flurbiprofen administered through the subglottic port of tracheal tubes to prevent POST in cardiac surgery
Schachtel et al., 2023 [20]	Total = 24; flurbiprofen = 11, placebo = 13	N/A	N/A	N/A	>18	N/A	Streptococcal pharyngitis	Flurbiprofen 8.75 mg lozenge	Placebo	Efficacy of flurbiprofen lozenge before and concomitant with antibiotics in Streptococcal pharyngitis
Muderris et al., 2019 [24]	Total = 136; flurbiprofen = 46, benzydamine = 45, placebo = 45	23 (flurbiprofen), 22 (benzydamine), 26 (placebo)	23 (flurbiprofen), 23 (benzydamine), 19 (placebo)	38.9 (flurbiprofen), 34.49 (benzydamine), 38.2 (placebo)	18	60	POST	Flurbiprofen 1.3 mg spray	Benzydamine hydrochloride and Placebo	Effectiveness of preoperative flurbiprofen in alleviating POST and hoarseness
Schachtel et al., 2018 [12]	Total = 122; flurbiprofen = 101, placebo = 21	Total = 51; flurbiprofen = 43, placebo = 8	Total = 71; flurbiprofen = 58, placebo = 13	Total = 19.5; flurbiprofen = 19.5, placebo = 19.6	18	33	Acute pharyngitis due to URTI	Flurbiprofen 8.75 mg lozenge	Placebo	Determining the onset of analgesia by flurbiprofen using a DSW method
Schachtel et al., 2018 [14]	equal to [12]									Evaluation of QuaSTI in measuring descriptors of sore throat
Burova et al., 2018 [13]	Total = 439; spray = 217, lozenge = 222	Total = 180; spray = 85, lozenge = 95	Total = 259; spray = 132, lozenge = 127	Total = 41.9; spray = 41.6, lozenge = 42.1	18	75	Acute sore throat due to URTI	Flurbiprofen 8.75 mg spray	Flurbiprofen 8.75 mg lozenge	Speed of relief and improvement of throat function with flurbiprofen
Radkova et al., 2017 [16]					equal to [13]					Efficacy of flurbiprofen spray or lozenge in patients
de Looze et al., 2016 [19]	Total = 498; flurbiprofen = 244, placebo = 254	Total = 283; flurbiprofen = 145, placebo = 138	Total = 222; flurbiprofen = 104, placebo = 118	Total = 25.6; flurbiprofen = 25.5, placebo = 25.7	18	73 (67 for flurbiprofen)	Acute sore throat due to URTI	Flurbiprofen 8.75 mg spray	Placebo	Efficacy and safety of flurbiprofen 8.75 mg spray
de Looze et al., 2018 [15]	equal to [19]									Efficacy of flurbiprofen spray for sore throat relief
Schachtel et al., 2016 [17]	Total = 204; flurbiprofen = 102, placebo = 102	87	117	19.8	18	26	Acute pharyngitis due to URTI	Flurbiprofen 8.75 mg lozenge	Placebo	Multiple doses of flurbiprofen lozenges for relief of pain, difficulty swallowing and swollen throat
Aspley et al., 2016 [18]	Total = 124; flurbiprofen = 59, placebo = 65	Total = 57; flurbiprofen = 34, placebo = 23	Total = 67; flurbiprofen = 25. placebo = 42	Total = 19.7; flurbiprofen = 19.8, placebo = 19.6	18	26 (24 for placebo)	Acute pharyngitis due to URTI	Flurbiprofen 8.75 mg lozenge	Placebo	Efficacy and safety of flurbiprofen 8.75 mg lozenge in adults with a swollen and inflamed throat

DSW: Double Stopwatch; QuaSTI: Qualities of Sore Throat Index; N/A: Not Applicable

**Table 4:** An overview of the study cohort in the included review papers.

Studies	Number of Included Studies (*n*)	Total Number of Subjects (*n*)	Intervention	Aim
Abdullah et al., 2024 [1]	32	N/A	Topical flurbiprofen	To improve diagnostic accuracy, decrease unwarranted antibiotic prescriptions and enhance patient outcomes in acute sore throat
Evans et al., 2024 [21]	26	2995	Flurbiprofen 8.75 mg	Risk of adverse effects (excluding hemorrhagic) resulting from drug–drug interactions
Dhanda et al., 2021 [22]	3	414	Flurbiprofen 8.75 mg	To identify existing evidence on the risk of hemorrhagic events with flurbiprofen 8.75 mg
de Looze et al., 2019 [23]	17	3848	Topical flurbiprofen 8.75 mg	To review the available clinical evidence for the efficacy and safety of topical 8.75 mg flurbiprofen for the symptomatic management of pharyngitis/sore throat

**Table 5:** An overview of the scales and questionnaires used in the included studies.

Assessment Tool	Description
**Pain**	
STPIS (Sore Throat Pain Intensity Scale) [23]	100 mm VAS with 0 = no pain and 100 = severe pain
STS (Sore Throat Scale) [23]	11-point scale with 0 = not sore and 10 = very sore
STRRS (Sore Throat Relief Rating Scale) [23]	Seven-point categorical scale: no, slight, mild, moderate, considerable, almost complete and complete relief
TSS (Throat Soreness Scale) [23]	11-point scale with 0 = not sore and 10 = very sore
TPS (Throat Pain Scale) [4]	Four-point verbal scale with 0 = no sore throat and 3 = severe (more than a common cold)
VAS (Visual Agonal Scale) [4]	100 mm with 0 = no pain and 100 = the worst imaginable pain
DSW (Double Stopwatch) Method [12]	Subjects instructed to depress the stopwatch when they perceive any pain relief and a second stopwatch when they experience subjective meaningful pain relief
**Difficulty swallowing**	
DSS (Difficulty Swallowing Scale) [23]	100 mm VAS (Visual – Analog Scale) with 0 = not difficult and 100 = very difficult
**Throat swelling**	
SwoTS (Swollen Throat Scale) [23]	100 mm VAS with 0 = not swollen and 100 = very swollen
**URTI assessment**	
URTI (Upper Respiratory Tract Infection) Questionnaire [14,23]	A questionnaire assessing the presence or absence of 39 different symptoms of URTI
**Overall assessment**	
SATIS (Patient Satisfaction Scale) [23]	Seven-point categorical scale: extremely dissatisfied, very dissatisfied, dissatisfied, somewhat satisfied, satisfied, very satisfied, extremely satisfied
GLOBAL (Patient’s Global Evaluation of The Study Treatment) [23]	Five-point categorical scale: poor, fair, good, very good, excellent
CLIN (Practitioner’s Clinical Assessment of Drug Efficacy) [16]	Five-point categorical scale: poor, fair, good, very good, excellent
**Other**	
QuaSTI (Qualities of Sore Throat Index) [23]	11-item composite index comprising the STS score and 10 other qualities of sore throat assessed on a 0–10 Likert scales (Factor 1: burning, raw, dry, husky/hoarse voice, irritated/scratchy; Factor 2: like a lump in the throat, tight, difficulty swallowing, swollen, soreness; Factor 3: agonizing)
HOAR (Hoarseness, Roughness, Breathiness, Asthenia) scale [4]	Four-point scale with 0 = no hoarseness and 3 = severe hoarseness

**Table 6:** An overview of the results of flurbiprofen on pain levels/relief in the included trials and reviews, divided by types of studies, interventions and doses.

Reference	Intervention	Outcome Measure	Key Outcomes
**Flurbiprofen vs. placebo**			
**Single dose**			
Schachtel et al., 2018 [12]	Flurbiprofen 8.75 mg lozenge or placebo	DSW (Double Stopwatch in mm)	Median time to meaningful pain relief was 43 min in the flurbiprofen group (95% CI (Confidence Interval): 36.4–49.4 min, *p* = 0.01) First perceived pain relief was 11 min in the flurbiprofen group, 19 min in the placebo (*p* = 0.03) Meaningful pain relief was reported by 78% in the flurbiprofen group and 48% in the placebo in 3 h (*p* < 0.01)
		STPIS (Sore Throat Pain Intensity Scale in mm)	Mean reduction at 43 min of 42% in the flurbiprofen group was not significantly different from the 48% in the placebo group (*p* = 0.38)
		STS (Sore Throat Scale)	Mean 2.2 reduction from baseline at 45 min in the flurbiprofen group and 1.2 for the placebo group
de Looze et al., 2018 [15]	Flurbiprofen 8.75 mg spray or placebo	STRRS (Swollen Throat Soreness Scale)	Reduction of ≥ −2.2 from 75 min to 6 h Mean TOTPAR (Total Sum of Pain Relief Ratings) 0–6h was significantly greater with flurbiprofen than placebo (3.24 vs. 2.47, *p* < 0.0001) The peak number of patients with at least moderate relief was at 180 min (46% flurbiprofen, 27% placebo) The maximum difference between the groups was at 75 min (38% flurbiprofen, 19% placebo)
Aspley et al., 2016 [18]	Flurbiprofen 8.75 mg lozenge or placebo	STPIS (mm)	Reduction in sore throat pain with flurbiprofen compared with placebo lozenge from 22 to 210 min (*p* < 0.05)
de Looze et al., 2016 [19]	Flurbiprofen 8.75 mg spray or placebo	TSS	AUC (area under the change from baseline curve) 0–2h, 0–3h and 0–6h were significantly greater with flurbiprofen spray than placebo (*p* < 0.0001) The change in severity of throat soreness was greater with flurbiprofen than placebo from 5 min to 6 h (*p* < 0.01)
		STPIS (mm)	STPIS had a significantly greater reduction than placebo after 20 min (*p* < 0.01)
		STRRS	STRRS had a greater change from baseline than placebo from 20 min to 6 h (*p* < 0.0001) At least 30 min of at least moderate relief in 6 h was reported in 55% of those treated with flurbiprofen and 34.8% of those treated with placebo (*p* < 0.001)
**Multiple doses**			
Schachtel et al., 2016 [17]	Flurbiprofen 8.75 mg lozenge or placebo	STPIS (mm)	Mean SPID 24 h (time-weighted summed difference in sore throat pain intensity) −437.7 mm/h with flurbiprofen and −322.3 mm/h with placebo (47% greater reduction, *p* < 0.05) Overall 74% greater sore throat relief with flurbiprofen than placebo from before to 2 h after each dose in days 2–7 (−13.9mm vs. −8.0mm, *p* < 0.01)
Aspley et al., 2016 [18]	Flurbiprofen 8.75 mg lozenge or placebo	STPIS (mm)	Mean relief of sore throat pain was 79.8% greater with flurbiprofen after 24 h (−477.8 mm*h and −265.7 mm*h, *p* < 0.01)
de Looze et al., 2016 [19]	Flurbiprofen 8.75 mg spray or placebo	TSS	Greater reduction in the change from baseline with flurbiprofen than placebo at the end of days 1–3 (all *p* < 0.05)
		STPIS (mm)	
		STRRS	
**Flurbiprofen spray vs. lozenge**			
Burova et al., 2018 [13]	Flurbiprofen 8.75 mg spray or flurbiprofen 8.75 mg lozenge	STRRS	‘At least moderate relief’ at 1 min post-dose and 74–78% at 2 h post-dose reported by 55–59% (no significant difference between the groups *p* > 0.05) TOTPAR was not significantly different between the groups (2.55 spray and 2.57 lozenge, pp = 0.7305)
Radkova et al., 2017 [16]	Flurbiprofen 8.75 mg spray or flurbiprofen 8.75 mg lozenge	STPIS PID (mm)	STPIS 2 h mean −40.51 in the spray and −40.10 in the lozenge (*p* = 0.83, 95% CI)
**Flurbiprofen and antibiotics**			
Schachtel et al., 2023 [20]	Flurbiprofen 8.75 mg lozenge or placebo and antibiotic	STPIS (mm)	Mean relief was 93% greater with flurbiprofen versus placebo (−10.9 vs. −5.7, *p* = 0.05) 24 h before antibiotic administration Mean relief was 84% greater with flurbiprofen versus placebo (−11 vs. −6, *p* = 0.04) during antibiotic administration The outcomes were not influenced by antibiotics in the flurbiprofen group (*p* = 0.96) and in the placebo (*p* = 0.8)
**Reviews**			
de Looze et al., 2019 [23]	Flurbiprofen 8.75 mg locally	STPIS (mm), STRRS, TSS, STS	Overall, flurbiprofen 8.75 mg provided significant relief of sore throat pain and soreness compared with placebo (single and multiple doses)
Abdullah et al., 2024 [1]	Flurbiprofen	N/A (Not Applicable)	NSAIDs can give relief from the symptomatic pain of acute sore throat (100% degree of agreement) Improvement of 59% in throat pain with multiple doses of flurbiprofen lozenge across 24 h compared with placebo.

**Table 7:** An overview of the results of flurbiprofen on difficulty swallowing in the included trials and reviews, divided by types of studies, interventions and doses.

Reference	Intervention	Outcome Measure	Key Outcomes
**Flurbiprofen vs. placebo**			
**Single dose**			
Aspley et al., 2016 [18]	Flurbiprofen 8.75 mg lozenge or placebo	DSS (Difficulty Swallowing Scale in mm)	Statistically significant reduction in difficulty swallowing after a single dose of flurbiprofen from 10 to 210 min compared with placebo (*p* < 0.05)
de Looze et al., 2016 [19]	Flurbiprofen 8.75 mg spray or placebo	DSS (mm)	DSS AUC (Area Under Curve) 0–2h, 03h, 0–6h were significantly in favor of flurbiprofen over placebo (*p* < 0.0001) Difficulty swallowing had a significant change from baseline compared to placebo from 5 min (*p* < 0.05) for up to 6 h
**Multiple doses**			
Schachtel et al., 2016 [17]	Flurbiprofen 8.75 mg lozenge or placebo	DSS (mm)	Improvement in swallowing was 66% greater with flurbiprofen than placebo after 24 h (mean DSS 24 −458.4 mm*h for flurbiprofen and −276.7 mm*h for placebo, *p* < 0.01) Mean improvement in swallowing was 72% greater with flurbiprofen than placebo before and 2 h post-dose in days 2 to 7 (−14.1 mm for flurbiprofen and −8.2mm for placebo, *p* < 0.01)
Aspley et al., 2016 [18]	Flurbiprofen 8.75 mg lozenge or placebo	DSS (mm)	Mean relief of difficulty swallowing was 99.6% greater with flurbiprofen than placebo over 24 h (−441.0 mm*h with flurbiprofen and −220.9 mm*h with placebo, *p* < 0.01)
de Looze et al., 2016 [19]	Flurbiprofen 8.75 mg spray or placebo	DSS (mm)	Greater reduction in change from baseline in the severity of difficulty swallowing with flurbiprofen than placebo at the end of days 1, 2 and 3 (*p* < 0.05)
**Flurbiprofen spray vs. lozenge**			
Burova et al., 2018 [13]	Flurbiprofen 8.75 mg spray or flurbiprofen 8.75 mg lozenge	DSS (mm)	Scores for difficulty swallowing improved in both groups at 1 h and 2 h post-dose compared with baseline, with no significant LS mean difference between the groups (*p* = 0.77) No significant difference between the groups in LS means DSS AUC 0-2h (*p* = 0.37)
**Flurbiprofen and antibiotics**			
Schachtel et al., 2023 [20]	Flurbiprofen 8.75 mg lozenge or placebo and antibiotic	DSS (mm)	Reduction in difficulty swallowing was 71% greater with flurbiprofen than placebo 24 h before antibiotic administration but not statistically significant (*p* = 0.16) Reduction in difficulty swallowing was 107% greater with flurbiprofen than placebo during antibiotic coadministration (*p* = 0.04) No significant relief was found with antibiotics on difficulty swallowing
**Reviews**			
de Looze et al., 2019 [23]	Flurbiprofen 8.75 mg locally	DSS (mm)	Flurbiprofen resulted in a relief of difficulty swallowing in single and multiple doses
Abdullah et al., 2024 [1]	Flurbiprofen	N/A (Not Applicable)	Flurbiprofen lozenge in multiple doses across 24 h lead to a 45% decrease in the level of difficulty swallowing compared with placebo

**Table 8:** An overview of the results of flurbiprofen on throat swelling in the included trials and reviews, divided by types of studies, interventions and doses.

Reference	Intervention	Outcome Measure	Key Outcomes
**Flurbiprofen vs. placebo**			
**Single dose**			
Aspley et al., 2016 [18]	Flurbiprofen 8.75 mg lozenge or placebo	SwoTS (Swollen Throat Scale - mm)	Greater relief of throat swelling with flurbiprofen than placebo was noted from the first assessment (60 min) through 180 min (*p* < 0.05)
de Looze et al., 2016 [19]	Flurbiprofen 8.75 mg spray or placebo	SwoTS (mm)	Changes from baseline in SwoTS AUC at 2 h, 3 h and 6 h in favor of flurbiprofen (*p* < 0.0001) Change from baseline in severity of throat swelling was significantly greater with flurbiprofen than placebo from 30 min to 6 h (*p* < 0.001)
**Multiple doses**			
Schachtel et al., 2016 [17]	Flurbiprofen 8.75 mg lozenge or placebo	SwoTS (mm)	Reduction in the sensation of swollen throat was 40% greater (mean SwoTS 24) compared with placebo over 24 h (−482.4 mm*h with flurbiprofen and −344.8 mm*h with placebo, *p* = 0.04) Reduction in swollen throat was 63% greater with flurbiprofen than placebo before and 2 h post-dose in days 2–7 (−15.0 mm for flurbiprofen and −9.2 mm for placebo, *p* < 0.01)
Aspley et al., 2016 [18]	Flurbiprofen 8.75 mg lozenge or placebo	SwoTS (mm)	Mean reduction in the sensation of swollen throat was 69.3% greater with flurbiprofen than placebo over 24 h (501,7 mm*h with flurbiprofen and 296.4 with placebo, *p* < 0.05)
de Looze et al., 2016 [19]	Flurbiprofen 8.75 mg spray or placebo	SwoTS (mm)	Greater reduction in change from baseline in the severity of throat swelling with flurbiprofen than placebo at the end of days 1 and 2 (*p* < 0.05) At the end of day 3, the reduction was not statistically significant (*p* = 0.147)
**Flurbiprofen spray vs. lozenge**			
Burova et al., 2018 [13]	Flurbiprofen 8.75 mg spray or flurbiprofen 8.75 mg lozenge	SwoTS (mm)	Swollen throat scores improved in both groups after 1 and 2 h post-dose (LS mean −26.64 (spray), −25.57 (lozenge) at 1 h, −30.51 (spray), −29.48 (lozenge) at 2 h) The difference in LS mean between groups was not significant at 1h (*p* = 0.443) or at 2 h (*p* = 0.498) No significant difference between spray and lozenge at LS means SwoTS AUC 0–2h (*p* = 0.391)
**Flurbiprofen and antibiotics**			
Schachtel et al., 2023 [20]	Flurbiprofen 8.75 mg lozenge or placebo and antibiotic	SwoTS (mm)	Reduction in the sensation of swollen throat was 295% greater for the flurbiprofen group than placebo (LS mean −8.7 mm with flurbiprofen and −2.2 with placebo, *p* = 0.008) Relief of throat swelling was 70% greater with flurbiprofen than placebo during antibiotic coadministration (LS mean −9.7 mm with flurbiprofen and -5.7 mm with placebo, *p* = 0.06—no significant difference) SwoTS scores were not influenced by antibiotics in the flurbiprofen group (*p* = 0.54) but were in the placebo group (61% greater reduction, = 0.02)
**Reviews**			
de Looze et al., 2019 [23]	Flurbiprofen 8.75 mg locally	SwoTS (mm)	The data generally support flurbiprofen as being effective in reducing the sensation of a swollen throat following single and multiple doses (1–7 days)
Abdullah et al., 2024 [1]	Flurbiprofen lozenge	N/A (Not Applicable)	Flurbiprofen in multiple doses across 24 h lead to a 44% reduction in throat swelling compared with placebo

**Table 9:** An overview of the results of flurbiprofen on the QuaSTI score in the included trials and reviews, divided by types of studies, interventions and doses.

Reference	Intervention	Outcome Measure	Key Outcomes
**Flurbiprofen vs. placebo**			
**Single dose**			
Schachtel et al., 2018 [14]	Flurbiprofen 8.75 mg lozenge or placebo	QuaSTI (Qualities of Sore Throat Index)	Change from baseline in the overall score was significant for the flurbiprofen group (31%) but not for placebo (11%) (*p* < 0.01) The mean change from baseline for each quality of pain except ‘burning’ and ‘like a lump in the throat’ was significantly greater with flurbiprofen (*p* ≤ 0.5)
**Flurbiprofen spray vs. lozenge**			
Burova et al., 2018 [13]	Flurbiprofen 8.75 mg spray or flurbiprofen 8.75 mg lozenge	QuaSTI	The mean change from baseline showed improvement for each individual score for both formulations 2 h post-dose (all *p* < 0.0001) The mean improvement from baseline to 2 h post-dose for all items showed no difference between formulations (*p* < 0.0001)
**Reviews**			
de Looze et al., 2019 [23]	Flurbiprofen 8.75 mg locally	QuaSTI	Overall QuaSTI scores improved from baseline after the use of flurbiprofen spray or lozenge compared with placebo

**Table 10:** An overview of the results of flurbiprofen on the overall satisfaction scores in the included trials and reviews, divided by types of studies, interventions and doses.

Reference	Intervention	Outcome Measure	Key Outcomes
**Flurbiprofen vs. placebo**			
**Single dose**			
de Looze et al., 2018 [15]	Flurbiprofen 8.75 mg spray or placebo	SATIS (Sore Throat Pain Intensity Scale)	In this study, 53.2% of patients taking flurbiprofen reported being at least ‘’satisfied’’ (SATIS ≥ 5) 3 h post-dose compared with 32.7% taking placebo (*p* < 0.0001)
Schachtel et al., 2016 [17]	Flurbiprofen 8.75 mg lozenge or placebo	GLOBAL (Global Evaluation)	In this study, 54% of patients treated with flurbiprofen rated the treatment at least ‘good’’ 2 h after dosing compared with 27% of placebo (*p* < 0.01)
**Multiple doses**			
de Looze et al., 2018 [15]	Flurbiprofen 8.75 mg spray or placebo	SATIS	In this study, 55.9% of patients taking flurbiprofen reported being at least ‘satisfied’ (SATIS ≥ 5) at the end of day 3 compared with 43% taking placebo (*p* < 0.01)
Schachtel et al., 2016 [17]	Flurbiprofen 8.75 mg lozenge or placebo	SATIS	In this study, 47% of patients treated with flurbiprofen were at least ‘’satisfied’’ over 24 h compared with 28% of the placebo group (*p* < 0.01)
		GLOBAL	In this study, 59% of patients treated with flurbiprofen rated the treatment at least ‘good’’ 24 h after dosing compared with 39% of placebo (*p* < 0.01)
		CLIN (Clinical Assessment of the medication as a treatment for sore throat)	In this study, 50% of treatments with flurbiprofen and 34% of treatments with placebo were rated as at least ‘’good’’ 24 h after dosing (*p* < 0.01) Furthermore, 60% of treatments with flurbiprofen were rated as at least ‘’good’’ and 34% of treatments with placebo after 7 days of treatment (*p* < 0.01)
de Looze et al., 2016 [19]	Flurbiprofen 8.75 mg spray or placebo	SATIS	N/R
		GLOBAL	In this study, 64.3% of patients treated with flurbiprofen rated it as ‘’good’’ or above compared with 49.8% of patients treated with placebo at the end of day 3 (*p* < 0.05)
		CLIN	In this study, 55.6% of practitioners rated treatment with flurbiprofen as ‘’good’’ or above compared with 44.7% for the placebo treatment at the end of day 3 (*p* < 0.05)
**Flurbiprofen spray vs. lozenge**			
Burova et al., 2018 [13]	Flurbiprofen 8.75 mg spray or flurbiprofen 8.75 mg lozenge	GLOBAL	In this study, 81% of patients in the spray group and 74% in the lozenge group rated the treatment as at least ‘’good’’ 2 h post-dose There was no significant difference between the groups at 2 h post-dose (*p* = 0.4007)
Radkova et al., 2017 [16]	Flurbiprofen 8.75 mg spray or flurbiprofen 8.75 mg lozenge	SATIS	No significant difference between the groups at 2 h post-dose; In this study, 89% (spray) and 84% (lozenge) of patients reported they were at least ‘’satisfied’’ at 2 h post-dose
		CLIN	No significant difference between the groups at 2 h post-dose In this study, 86% (spray) and 70% (lozenge) were reported as at least ‘’good’’ at 2 h post-dose
**Reviews**			
de Looze et al., 2019 [23]	Flurbiprofen 8.75 mg locally	SATIS	Significantly more patients taking flurbiprofen stated that they were at least ‘’satisfied’’ compared to placebo with no significant difference between spray and lozenge
		GLOBAL	Significantly more patients taking flurbiprofen rated the treatment as at least ‘’good’’ compared to placebo with no significant difference between spray and lozenge

**Table 11:** An overview of the results of flurbiprofen on the URTI questionnaire in the included trials and reviews, divided by types of studies, interventions and doses.

Reference	Intervention	Outcome Measure	Key Outcomes
**Flurbiprofen vs. placebo**			
**Single dose**			
Schachtel et al., 2018 [14]	Flurbiprofen 8.75 mg lozenge or placebo	URTI (Upper Respiratory Tract Infection)	A significant number of patients treated with flurbiprofen reported no achiness, pressure under the eyes, mouth breathing, lack of energy, tender neck glands, headache, loss of appetite, sinus pressure, coughing, chest tightness or sinus pain 3 h post-dose (all *p* < 0.05) Of patients with coughing before flurbiprofen administration, 46% reported no coughing 3 h post-dose (*p* = 0.01) There was no significant change from baseline between these other URTI symptoms in placebo-treated patients
**Flurbiprofen spray vs. lozenge**			
Burova et al., 2018 [13]	Flurbiprofen 8.75 mg spray or flurbiprofen 8.75 mg lozenge	URTI	The number of patients experiencing ST-related URTI symptoms decreased 2 h post-dose in both formulations, except for tender neck glands and throat tickle A percentage of patients developed some URTI symptoms in the 2 h period (throat tickle, throat clearing)
**Reviews**			
de Looze et al., 2019 [23]	Flurbiprofen 8.75 mg locally	URTI	Flurbiprofen was shown to significantly reduce URTI symptoms compared with placebo

**Table 12:** An overview of the results of flurbiprofen on the incidence and severity of POST in the included trials and reviews, divided by types of studies, interventions and doses.

Reference	Intervention	Outcome Measure	Key Outcomes
Calabrese et al., 2024 [4]	Flurbiprofen 0.25% solution in subglottic port of the endotracheal tube	VAS (Visual – Analog Scale in mm)	In this study, 5.9% of the flurbiprofen group and 100% of the placebo group at 15 min and 3 h reported POST Median VAS 15 min, 3 h, 12 h, 36 h was 0 in the flurbiprofen group and 45, 35, 20, 10 in the placebo group (all *p* < 0.001)
		TPS (Throat Pain Scale)	Median TPS 15 min, 3 h, 12 h, 36 h was 0 in the flurbiprofen group and 2, 2, 1, 1 in the placebo group (all *p* < 0.001)
		HOAR (Hoarseness, Roughness, Breathiness, Asthenia)	In this study, 5.9% of the flurbiprofen group and 97% of the placebo group reported hoarseness at 15 min and 3 h Median HOAR 15 min, 3 h, 12 h, 36 h was 0 in the flurbiprofen group and 2, 1, 1, 0 in the placebo group (all *p* < 0.001)
		SATIS (Sore Throat Pain Intensity Scale)	Patients in the flurbiprofen group reported significantly higher levels of satisfaction than placebo (*p* < 0.001)
Muderris et al., 2019 [24]	Flurbiprofen 1.3 mg spray or benzydamine hydrochloride 1.08 mg spray or placebo	4-point POST (Postoperative Sore Throat) scale	The incidence of sore throat was significantly lower for flurbiprofen (17.3%) than placebo (37.7%) The mean severity scores were significantly lower in treatment groups compared to placebo at all time points (20 min, 1 h, 6 h, 24 h)
		4-point hoarseness scale	The incidence of hoarseness was significantly lower with flurbiprofen (8.6%) than placebo (24.4%) The level of hoarseness was significantly lower in treatment groups than placebo at all time points (20 min, 1 h, 6 h, 24 h)
**Reviews**			
de Looze et al., 2019 [23]	Flurbiprofen 8.75 mg lozenge	N/A (Not Applicable)	Flurbiprofen was found to reduce the incidence and severity of POST in the early postoperative period

**Table 13:** An overview of the safety profile of flurbiprofen in the included trials and reviews, divided by types of studies, interventions and doses.

Reference	Intervention	Incidence of any Adverse Effects	Reported Adverse Effects Related to Flurbiprofen	Adverse Effect Leading to Discontinuation
**Flurbiprofen vs. placebo**				
**Single dose**				
Schachtel et al., 2018 [12], Schachtel et al., 2018 [14]	Flurbiprofen 8.75 mg lozenge or placebo	9% of patients (*n* = 11, *n* (flurbiprofen) = 10)	Abdominal discomfort and throat irritation (2%)	None
de Looze et al., 2016 [19], de Looze et al., 2018 [15]	Flurbiprofen 8.75 mg spray or lozenge	6.8% of patients with flurbiprofen, 3.1% of patients with placebo (*p* = 0.055)	No adverse effects were assessed as definitely related to flurbiprofen	None related to flurbiprofen
**Multiple doses**				
Schachtel et al., 2016 [17]	Flurbiprofen 8.75 mg lozenge or placebo	35.3% of patients with flurbiprofen, 32.4% of patients with placebo (*p* = 0.66)	Reported in 19.6% of patients with flurbiprofen, 7.8% with placebo (*p* = 0.01) Most commonly reported were stomatitis, oral pain, oral paresthesia, abdominal pain and nausea, tonsillitis and throat irritation, otitis media and headache No significant difference between groups in gastrointestinal symptoms All AEs resolved without sequelae	None related to the administered drugs (two taking flurbiprofen and three taking placebo)
Aspley et al., 2016 [18]	Flurbiprofen 8.75 mg lozenge or placebo	25.4% of patients taking flurbiprofen, 27.7% taking placebo (*p* = 0.775)	Not directly assessed	None
de Looze et al., 2016 [19]	Flurbiprofen 8.75 mg spray or lozenge	12.4% of patients with flurbiprofen, 8.2% of patients with placebo at the end of day 3 (*p* = 0.119)	No adverse effects were assessed as definitely related to flurbiprofen	None
**Flurbiprofen spray vs. lozenge**				
Radkova et al., 2017 [16], Burova et al., 2018 [13]	Flurbiprofen 8.75 mg spray or flurbiprofen 8.75 mg lozenge	44% for the spray (*n* = 96), 35.6% for the lozenge (*n* = 79)	Spray (4.1%, *n* = 9): throat irritation, dyspepsia, malaise, cough, hiccups Lozenge (1.8%, *n* = 4): glossodynia, tachycardia, dyspepsia, hypoesthesia and somnolence	None
**POST**				
Calabrese et al., 2024 [4]	Flurbiprofen 0.25% solution	None	None	None
Muderris et al., 2019 [24]	Flurbiprofen 1.3 mg spray or benzydamine hydrochloride 1.08 mg spray or placebo	2.9% of patients in all three groups (*n* = 0) for flurbiprofen	None	None
**Reviews**				
Evans et al., 2024 [21]	Flurbiprofen 8.75 mg (any formulation)	N/R	In total, 14/26 studies reported ≥ 1 drug-related AE (Adverse Effects) Taste perversion, paresthesia, headache, dizziness, nausea, dyspepsia, diarrhea, dry mouth, dry throat, abdominal pain, abdominal discomfort, dry nipping throat, throat irritation, cough, hiccups, glossodynia, hypoesthesia, somnolence, malaise, tachycardia, GI (Gastrointestinal) adverse effects were reported No AEs were reported as a result of drug interactions	N/R
Dhanda et al., 2021 [22]	Flurbiprofen 8.75 mg (any formulation)	≥1% of hemorrhagic event with flurbiprofen 8.75 mg	N/R	N/R
de Looze et al., 2019 [23]	Flurbiprofen 8.75 mg locally	N/R	Similar between flurbiprofen and placebo groups Most commonly GI (nausea, dyspepsia, diarrhea, abdominal pain/discomfort) or nervous system (dry mouth, paresthesia, throat irritation)	Related to underlying medical conditions or subsequently not considered to be of clinical relevance
Abdullah et al., 2024 [1]	Flurbiprofen topically	N/R	The use of topical/localized NSAID (Non-Steroid Anti-Inflammatory Drugs) in the treatment of pain in acute sore throat poses better benefit–risk ratio compared to oral NSAIDs (100% degree of agreement) GI AEs and risk of renal failure are not induced by topical NSAID (lozenge or spray)	N/A (Not Applicable)

## Data Availability

The original data presented in the study are openly available in OSF (https://osf.io/mcb6d, accessed on 23 October 2025).

## Supplementary Materials

The following supporting information can be downloaded at: https://www.mdpi.com/article/10.3390/biomedicines13123035/s1, Table S1: PRISMA 2020 Checklist, reference [30] is cited in Supplementary material.

## Abbreviations

The following abbreviations are used in this manuscript:

POST	Postoperative sore throat
AMR	Antimicrobial resistance
URTI	Upper respiratory tract infection
TPA	Tonsillo-Pharyngitis Assessment
STS	Sore Throat Scale
TSS	Throat soreness scale
TPS	Throat Pain Scale
STPIS	Sore Throat Pain Intensity Scale
DSS	Difficulty Swallowing Scale
SwoTS	Swollen Throat Scale
PAIN	Practitioner’s Assessment of Inflammation
STRRS	Sore Throat Relief Rating Scale
VAS	Visual Agonal Scale
DSW	Double Stopwatch
SATIS	Patient Satisfaction Scale
GLOBAL	Patient’s Global Evaluation of The Study Treatment
CLIN	Practitioner’s Clinical Assessment of Drug Efficacy
QuaSTI	Qualities of Sore Throat Index
TOTPAR	Total Pain Relief
AUC	Area Under the Change from Baseline Curve
NSAID	Non-steroidal Anti-inflammatory Drug
ICU	Intensive Care Unit
GI	Gastrointestinal
AE	Adverse Effect
SPID	The time-weighted summed difference in sore throat pain intensity
